# Counterfactual harm: a counter-argument

**DOI:** 10.1093/aje/kwag064

**Published:** 2026-03-19

**Authors:** Amit N Sawant, Mats J Stensrud

**Affiliations:** Institute of Mathematics, Ecole Polytechnique Fédérale de Lausanne, Lausanne, 1015, VD, Switzerland; Institute of Mathematics, Ecole Polytechnique Fédérale de Lausanne, Lausanne, 1015, VD, Switzerland

**Keywords:** ethical AI, harm, intransitivity, decision theory

## Abstract

As artificial intelligence systems are increasingly used to guide decisions, it is essential that they follow ethical principles. A core principle in medicine is nonmaleficence, often equated with “do no harm”. A formal definition of harm based on counterfactual reasoning has been proposed and popularized. This notion of harm has been promoted in simple settings with binary treatments and outcomes. Here, we highlight a problem with this definition in settings involving multiple treatment options. Illustrated by an example with 3 tuberculosis treatments (say, A, B, and C), we demonstrate that the counterfactual definition of harm can produce intransitive results: B is less harmful than A, C is less harmful than B, yet C is more harmful than A when compared pairwise. This intransitivity poses a challenge as it may lead to practical (clinical) decisions that are difficult to justify or defend. In contrast, an interventionist definition of harm based on expected utility forgoes counterfactual comparisons and ensures transitive treatment rankings.

## Introduction

The Hippocratic maxim of “First, do no harm” remains a principle in modern medicine. However, the question of what constitutes harm in a medical setting from an algorithmic perspective is unclear and has led to rich debate. For instance, Andrikiyan et al.^1^ stated that a considerable portion of medical advice given by artificial intelligence (AI)-powered chatbots was deemed “potentially harmful” by medical experts. A concrete definition of harm helps ensure that algorithm-based decision making also follows the principle of “doing no harm”.

Characterizing harm in mathematical terms has been variously attempted; see, eg, Sarvet and Stensrud^2^ for a review. In particular a counterfactual notion of harm has been considered,^3^^-^^6^ which requires comparison of joint (cross-world) counterfactuals. Sarvet and Stensrud^2^ also discussed an interventionist definition of harm, which is defined with respect to single-world quantities.^7^ All of these works, however, were considering the setting where treatments are binary.

Most existing work on counterfactual harm, but not interventionist harm, is further restricted to binary outcomes.^3^^-^^5^^,^^8^ With binary outcomes, Dawid and Senn, Sarvet and Stensrud^2^^,^^9^^,^^10^ discussed the interventionist philosophy and clarified that certain notions of harm, which were claimed to be impossible to express in interventionist terms^5^^,^^11^, have a clear interventionist formulation.

For many medical conditions, there are more than two treatment options. Also, outcomes of interest are not as simple as dichotomies. Diseases can get progressively worse without the patient dying. Patients may discontinue treatment due to unforeseen side-effects without being fully cured. Patients who initially received no treatment may be put on rescue treatment, without having a fatal outcome.

With nonbinary outcomes and singular treatments, results from the counterfactual definition of harm tend to be uninformative under plausible assumptions^12^^,^^13^; it is often not possible to determine from a counterfactual perspective whether a treatment is more beneficial than harmful^14^^-^^17^ without making strong, and in-principle untestable, cross-world assumptions.^7^

Regardless of whether counterfactual definitions of harm result in informative comparisons, subtle problems could arise. Illustrated by an example on tuberculosis (TB) treatment, we show that when we have more than 1 treatment, comparing treatments pairwise using the counterfactual definition of harm can lead to intransitivity in the total order of treatments. That is, if our objective is to minimize counterfactual harm, then a treatment $A=a_{2}$ is better than the baseline treatment $A=a_{1}$. Treatment $A=a_{3}$ is then better than treatment $A=a_{2}$ by the same criterion, but treatment $A=a_{3}$ is worse than treatment $A=a_{1}$ in a direct comparison, which is problematic. On the other hand, using the criterion of interventionist harm does not lead to intransitivity. Transitivity is often desirable. For example, transitivity is an axiom in the von Neumann–Morgenstern utility theorem^18^.

We start with basic definitions of counterfactual and interventionist harm, as discussed in detail in existing works.^2^^,^^3^^,^^6^

## Notions of harm

Consider a treatment $A$ that takes binary values $(A\in \{0, 1\})$ and an outcome of interest $Y$. For the sake of illustration, we will use discrete, ordinal outcomes, but our example can be readily adapted to continuous real-valued outcomes.

It is clear that some outcomes are always better than others. To be explicit, consider 3 outcomes as $y_{1}:$ death, $y_{2}:$ discontinuing treatment, and $y_{3}:$ being fully cured. Discontinuing treatment is preferable to death, even though it is a relatively worse outcome to being cured, meaning the outcomes can be ordered as $y_{1} < y_{2} < y_{3}$. Note, we overload the comparison operator ‘<’ to both compare 1 event or outcome to another, along with the conventional sense of comparing 2 numbers.

We can additionally define a utility function $\mu $ that assigns a specific real-valued utility to each outcome $Y$. The utility function is order-preserving so that if $y_{1} \leq y_{2}$, then $\mu (y_{1}) \leq \mu (y_{2})$. Without loss of generality, we assume that outcomes with a greater numerical utility are better.

For any individual $i$, there exist 2 counterfactual variables $Y_{i}^{a=1}$ and $Y_{i}^{a=0}$, which are respectively the potential outcomes if the individual were to be administered the treatment $(a=1)$ or not $(a=0)$. We omit the subscript $i$ where the interpretation is obvious.

We first describe the counterfactual definition of harm, which is commonly used in the literature.^3^^-^^6^^,^^15^ If the potential outcome under treatment is worse than the potential outcome under no treatment, then the treatment is counterfactually harmful for that individual. This can be expressed concisely using indicator functions as 


(1) \begin{align*}& \textrm{harm}_{i} = I(Y_{i}^{a=1} < Y_{i}^{a=0}).\end{align*}

Analogously, we can define the treatment to be beneficial for individual $i$ if 


(2) \begin{align*}& \textrm{benefit}_{i} = I(Y_{i}^{a=1}> Y_{i}^{a=0}).\end{align*}

Now suppose there is more than 1 treatment option. Let $\mathcal{A} = \{a_{1}, a_{2},\dots a_{n}\}$ be the set of all treatments available, including the possibility of no treatment. When there is more than 1 treatment, we define “doing no harm” against the existing standard-of-care. Suppose a treatment $A=a_{1}$ is the current standard-of-care. Another treatment $A=a_{2}$ is considered harmful for an individual if $Y^{a_{2}} < Y^{a_{1}}$. It is equivalent to say the treatment is harmful if $\mu (Y^{a_{2}}) < \mu (Y^{a_{1}})$.

It is impossible to observe more than 1 potential outcome $Y_{i}^{a}$ for any individual. Thus, evidence of counterfactual harm can only be identified at a population level, say, as $P(Y^{a_{2}} < Y^{a_{1}}) = \mathbb{E}[I(Y^{a_{2}} < Y^{a_{1}})]$, which is the probability of harm in the population.

It may be tempting to simplify the expression $P(Y^{a_{2}} < Y^{a_{1}})$ to $P(Y^{a_{2}} - Y^{a_{1}} < 0)$ but we should remember that outcomes $Y^{a}$ are defined to be events from an ordered set $\mathcal{Y}$. The sum or difference of 2 such $y$’s is ambiguous. In terms of utility however, we can indeed make sense of the statement $\mu (Y^{a_{2}}) - \mu (Y^{a_{1}}) < 0$ and consider its expectation over a population, since utilities are real-valued. Thus, we get an alternative definition of harm in terms of utility. A treatment $A=a_{2}$ is harmful compared to the standard-of-care $A=a_{1}$ if $\mathbb{E}[\mu (Y^{a_{2}}) - \mu (Y^{a_{1}})] = \mathbb{E}[\mu (Y^{a_{2}})] - \mathbb{E}[\mu (Y^{a_{1}})] < 0$.

Defining harm in terms of utility at the population level as opposed to individual outcomes has been termed as “interventionist harm”.^2^ We thus have 2 different definitions of harm as follows:

Definition 2.1. (Counterfactual harm). For a treatment $A=a_{2}$, counterfactual harm exists if $\mathbb{E}[I(Y^{a_{2}} < Y^{a_{1}})]> 0$, where $A=a_{1}$ is the existing standard-of-care.

Definition 2.2. (Interventionist harm). For a treatment $A=a_{2}$, interventionist harm exists if $\mathbb{E}[\mu (Y^{a_{2}})] -\mathbb{E}[\mu (Y^{a_{1}})] < 0$, where $A=a_{1}$ is the existing standard-of-care.

To assess if a treatment is harmful in the population, by either definition, we require functionals of the counterfactual distribution to be estimable. Specifically, consider a Randomized Control Trial (RCT) with 2 arms, the new treatment $A=a_{2}$ and the existing standard-of-care $A=a_{1}$. The marginal distribution of each counterfactual is identified in this trial, but the joint counterfactual distribution of $(Y^{a_{2}}, Y^{a_{1}})$ remains unidentified.

Interventionist harm is point-identified whenever individual treatment effects are point-identified and does not require consideration of the joint counterfactual distribution.^19^ On the other hand, we can use the marginal distributions to derive bounds for the probability of counterfactual harm. We now consider a simple situation with multiple treatment options for TB, and we will compare treatments using the 2 definitions of harm (Definitions 2.1, 2.2).

## An example on TB and intransitivity

Tuberculosis is an infectious disease that is prevalent particularly in South-East Asia. Most patients with a TB diagnosis have latent TB, which can progress to active disease if left untreated and even result in death.^20^

Effective TB treatment requires long-lasting medication courses of up to 8 months with a mixture of antibiotics.^20^ There are strong antibiotics which can cause side-effects such as nausea and vomiting,^21^ and also weak antibiotics. If there is incomplete eradication of the TB pathogen, due to inadequate compliance with the treatment or ineffective antibiotics, the patient develops drug-resistant TB which has a higher risk of progressing to active TB and death compared to drug-susceptible TB.

Thus, there is genuine concern that TB medication may be harmful. We describe a hypothetical scenario where pair-wise comparison of treatments to minimize harm gives an intransitive overall ordering. This example has been adapted from previous results on intransitive orderings.^22^^-^^24^

We consider outcomes $\mathcal{Y} = \{y_{1}, y_{2}, \ldots y_{6}\}$ that can be uncontroversially ordered as follows:

$y_{1}:$ Death from TB within 1 year.

$y_{2}:$ Extensively drug-resistant (XDR)-TB, resistant to strong antibiotics.

$y_{3}:$ Multiple drug-resistant (MDR)-TB, resistant to weak antibiotics.

$y_{4}:$ Latent TB.

$y_{5}:$ Fully cured of TB with some side-effects.

$y_{6}:$ Fully cured of TB without side-effects.

We define the utility function $\mu _{1}(Y)$ that simply outputs the numerical position of the outcome in the ordered set $\mathcal{Y}$ as the utility. If a patient dies of TB within 1 year, the outcome $y_{1}$ has utility equal to $1$, as it is the first element in $\mathcal{Y}$. The utility function $\mu _{1}$ is chosen for illustrative purposes and can be replaced with any other order-preserving utility function $\mu $.

### Initial diagnosis

Consider patients with a diagnosis of latent TB. If left untreated $(A=a_{1})$, one-sixth of such patients develop active disease and die within 1 year. The remaining five-sixths still have latent TB, which may progress to active disease later on. Thus, under the assumption of causal consistency^25^, which should be uncontroversial in our example, 


\begin{align*} P(Y^{a_{1}} = y_{1}) &= P(Y = y_{1} \mid A=a_{1}) = 1/6,\\ P(Y^{a_{1}} = y_{4}) &= P(Y = y_{4} \mid A=a_{1}) = 5/6. \end{align*}

**Figure 1:**
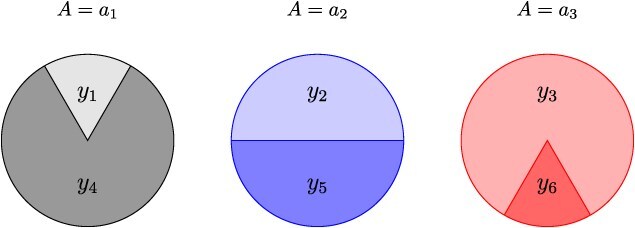
Pie charts representing potential outcomes $P(Y^{a}=y_{r})$ under “No Treatment” $(A=a_{1})$, “Strong Antibiotics” $(A=a_{2})$, and “Weak Antibiotics” $(A=a_{3}),$ respectively.

We can also depict this graphically as in Figure 1.

### Treatment with strong antibiotics

In an RCT (RCT-1) that compared strong antibiotics administered for 1 year $(A=a_{2})$ to no treatment $(A=a_{1})$, it was found that half of the patients in the treatment arm were fully cured of TB, and remarkably no patients in the treatment arm died of active disease. However, all of the patients experienced nausea throughout the course of medication, due to the strong immune response elicited by the antibiotics.

Owing to the side-effects and the long duration of treatment, half of the patients in the treatment arm developed XDR-TB due to incomplete adherence. Outcomes in the control arm under no treatment were the same as before. The results of the RCT can be summarized as follows,


\begin{align*} P(Y^{a_{2}} = y_{2}) &= P(Y = y_{2} \mid A=a_{2}) = 1/2, \\ P(Y^{a_{2}} = y_{5}) &= P(Y = y_{5} \mid A=a_{2}) = 1/2. \end{align*}

**Figure 2:**
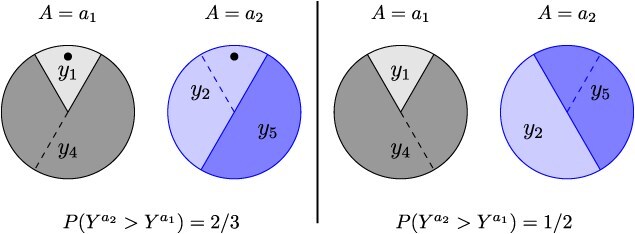
Joint potential outcomes for patients in RCT-1. Consider first the left set of pies, which shows a joint distribution of $(Y^{a_{1}}, Y^{a_{2}})$ that is compatible with RCT-1 and gives the maximum proportion of patients benefiting from treatment $A=a_{2}$ over $A=a_{1}$. The dot “•” indicates a patient with the 2 potential outcomes $Y^{a_{1}} = y_{1}, Y^{a_{2}} = y_{2}$, and it is understood that $P(Y^{a_{1}} = y_{1}, Y^{a_{2}} = y_{2}) = 1/6$. The right set of pies is also compatible with RCT-1, and gives the minimum proportion of patients benefiting from treatment $A=a_{2}$ over $A=a_{1}$; in particular $P(Y^{a_{1}} = y_{1}, Y^{a_{2}} = y_{2}) = 0$. Dashed lines are intended as guides for visual comparison and do not indicate actual partitions of the data.

Figure 2 shows the results of RCT-1 in graphical form. We can compute the probability of benefit and harm following Definition 2.1 based on the data from RCT-1. The procedure described here follows from classical Fréchet-Hoeffding bounds^26^^,^^27^ widely used in the literature on counterfactual harm.^3^^,^^4^^,^^13^^,^^14^ A short mathematical argument replicating the same is given in Appendix S1.

The left set of Figure 2 shows 1 possibility of the joint distribution of the 2 potential outcomes. All the patients who would have died under no treatment, would have survived with XDR-TB if given strong antibiotics treatment. Thus, this one-sixth of the population benefited from taking treatment $A=a_{2}$. In addition, half of the patients in the control arm, who would have survived with latent TB under no treatment, would have been fully cured with the antibiotics. Therefore, the maximum proportion of patients that would have benefited from the treatment is $1/6+1/2=2/3$.

The right set of Figure 2 represents the other extreme of the joint distribution. All the patients who would have died under no treatment, would be fully cured under treatment. In addition, one-third of the entire population, who would have survived with latent TB under no treatment, would have been fully cured with the antibiotics. Therefore, the minimum proportion of patients that would have benefited from the treatment is $1/2$.

In RCT-1 $P(\textrm{benefit}) = P(Y^{a_{2}}> Y^{a_{1}})$ is therefore bounded, 


(3) \begin{align*}& 1/2\ \leq\ P(Y^{a_{2}}> Y^{a_{1}})\ \leq \ 2/3.\end{align*}

With probability greater than or equal to half, the patient benefits from treatment $A=a_{2}$ over no treatment. Correspondingly, with at most probability $1/2$, the patient would be harmed by taking strong antibiotics. In addition, the edge case, $P(\textrm{benefit}) = P(\textrm{harm}) = 1/2$, occurs only if joint outcomes are strongly negatively correlated, ie, any patient with $Y^{a_{1}} = y_{1}$ always has $Y^{a_{2}} = y_{5}$. If even 1 patient in the population has the joint outcome $(Y^{a_{1}} = y_{1}, Y^{a_{2}} = y_{2})$, then the proportion of patients benefiting from strong antibiotics is strictly greater than $1/2$; see Appendix S1 for a rigorous proof.

From a perspective of maximizing benefit/minimizing harm in the counterfactual sense, it is clear that prescribing antibiotics is better than giving no treatment. We employ the simple decision rule that we switch to the new treatment if $P(\textrm{benefit}) \geq P(\textrm{harm})$. In Appendix S2, we consider decision rules that assign different weights to benefit and harm.

The expected utility under no treatment is $\mathbb{E}[\mu _{1}(Y^{a_{1}})] = 3.5$, and that under strong antibiotic treatment $\mathbb{E}[\mu _{1}(Y^{a_{2}})]$ is also $3.5$. The loss in utility for the patients who develop XDR-TB is offset by the gain in utility for the patients who are fully cured. Thus, there is no evidence of interventionist harm (or benefit) in this case.

Although there is a nonzero probability of counterfactual harm to the patient, it is impossible to know before prescribing medication whether the patient will be counterfactually harmed by treatment with antibiotics. Even if the patient has a worse outcome and would be considered “harmed” by this definition, the outcome of XDR-TB is better than dying, which is the worst outcome under no treatment.

We must take a call between prescribing no treatment and risking death of the patient, or prescribing antibiotics and risking XDR-TB. This is a tradeoff that exists in many practical settings. The utility function $\mu $ in the interventionist approach arguably serves to balance such tradeoffs. Some might reason that it is best to choose the treatment that avoids death as far as possible. To this end, we can enforce a utility function $\mu $ that maps ordered outcomes in $\mathcal{Y}$ to binary outcomes of death and survival. We consider utility functions other than $\mu _{1}$ in *Elaboration and explanation of the intransitivity* section.

### Treatment with weak antibiotics

Unpleasant side effects over the course of medication are undesirable. In another RCT (RCT-2), newer antibiotics $(A=a_{3})$ that did not cause side effects were compared to strong antibiotics $(A=a_{2})$ as the control.

In RCT-2, one-sixth of patients in the treatment arm were fully cured within 1 year, with no side-effects. The new antibiotics did not elicit a strong immune response however, so five-sixths of the patients ended up with MDR-TB. The results from the control arm under treatment $A=a_{2}$ were the same as those in RCT-1. The results of RCT-2 can be summarized as follows, 


\begin{align*} P(Y^{a_{3}} = y_{3}) &= P(Y = y_{3} \mid A=a_{3}) = 5/6, \\ P(Y^{a_{3}} = y_{6}) &= P(Y = y_{6} \mid A=a_{3}) = 1/6, \\ P(Y^{a_{2}} = y_{2}) &= P(Y = y_{2} \mid A=a_{2}) = 1/2, \\ P(Y^{a_{2}} = y_{5}) &= P(Y = y_{5} \mid A=a_{2}) = 1/2. \end{align*}

**Figure 3:**
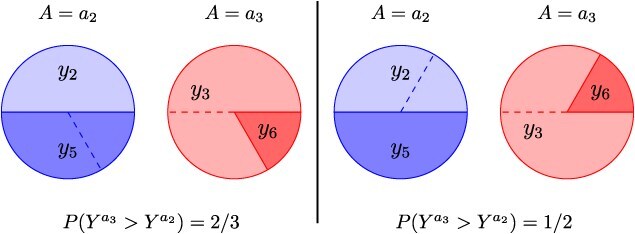
Maximum and minimum proportion of patients that can benefit from treatment $A=a_{3}$ over treatment $A=a_{2}$. As in Figure 2, dashed lines are intended as guides for visual comparison and do not indicate actual partitions of the data.

Figure 3 shows the results of RCT-2 in graphical form. As in the previous example (RCT-1), bounds on the potential benefit can be calculated.

In the left set of Figure 3, all of the patients who would have developed XDR-TB under strong antibiotics, would only develop MDR-TB under the new antibiotics. In addition one-sixth of the total population would be fully cured without side-effects under the new antibiotics, as opposed to being fully cured with side-effects under the old antibiotics. Therefore, the maximum proportion of patients that would have benefited from the new treatment over treatment with the older antibiotics is $1/6+1/2=2/3$.

Again, as in RCT-1, the lower bound for the probability of benefit is $1/2$, as depicted in the right set of figures in Figure 3. Thus, in RCT-2, $P(\textrm{benefit}) = P(Y^{a_{3}}> Y^{a_{2}})$ is bounded as


(4) \begin{align*}& 1/2\ \leq\ P(Y^{a_{3}}> Y^{a_{2}})\ \leq \ 2/3.\end{align*}

So, with at least probability half, the patient would benefit from taking the newer treatment $A=a_{3}$ over strong antibiotics, experiencing no side-effects. Except for strong determinisms in the population where $Y^{a_{2}} = y_{2}$ for every patient with $Y^{a_{3}} = y_{6}$, the probability of benefit is strictly bigger than $1/2$ (A rigorous argument is identical to the one for RCT-1 in Appendix S1). The probability of counterfactual harm if the patient is prescribed the new treatment is indeed nonzero, but even if the patient is “harmed”, the outcome of MDR-TB is still better than XDR-TB.

The expected utility under the new antibiotic treatment $\mathbb{E}[\mu _{1}(Y^{a_{3}})] = 3.5$ is also the same as for treatments $A=a_{1}$ or $A=a_{2}$ and there is no evidence of interventionist harm. So treatment with weak antibiotics is at least as good as treatment with strong antibiotics, given the objective of minimizing counterfactual harm.

### Interpreting the results of the 2 trials

Data from RCT-1 and RCT-2 can be combined to directly compare the new antibiotic treatment $(A=a_{3})$ and no treatment $(A=a_{1})$:


\begin{align*} P(Y^{a_{3}} = y_{3}) &= P(Y = y_{3} \mid A=a_{3}) = 5/6, \\ P(Y^{a_{3}} = y_{6}) &= P(Y = y_{6} \mid A=a_{3}) = 1/6, \\ P(Y^{a_{1}} = y_{1}) &= P(Y = y_{1} \mid A=a_{1}) = 1/6, \\ P(Y^{a_{1}} = y_{4}) &= P(Y = y_{4} \mid A=a_{1}) = 5/6. \end{align*}

**Figure 4:**
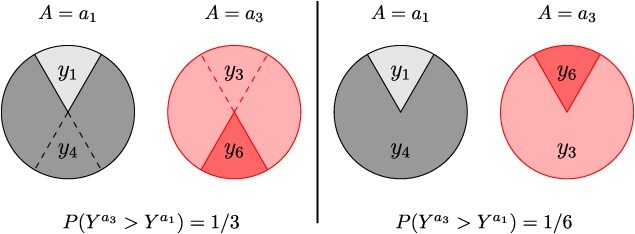
Maximum and minimum proportion of patients that can benefit from treatment $A=a_{3}$ over treatment $A=a_{1}$. Dashed lines serve purely as visual guides, same as Figures 2 and 3.

It is possible that the one-sixth of patients who would have died under no treatment are precisely the ones who get cured with the new antibiotics (rightmost pies in Figure 4). Thus, up to five-sixths of patients who would have latent TB under no treatment get MDR-TB after taking the new, weaker antibiotics. This argument is an intuitive justification for the following bounds, 


(5) \begin{align*}& 2/3\ \leq\ P(Y^{a_{1}}> Y^{a_{3}})\ \leq \ 5/6.\end{align*}

In summary, using the counterfactual definition, although strong antibiotics are as good as, or better than no treatment, and weak antibiotics are as good as, or better than strong antibiotics, weak antibiotics have a higher probability of potential harm compared to taking no treatment at all. In the 2 trials, corresponding to pair-wise comparisons, we minimized the probability of counterfactual harm yet we ended up with a treatment that is more harmful than the standard-of-care.

## Elaboration and explanation of the intransitivity

As we demonstrated, the objective of minimizing counterfactual harm is not one that guarantees a transitive ordering. One drawback of counterfactual harm is that the magnitude of difference between 2 outcomes is not taken into consideration, only whether a potential outcome is better or worse than the alternative. For example, in RCT-1, one-sixth of the patients benefit from taking treatment $A=a_{2}$, as they would have died if they were given no treatment. The magnitude of benefit in this case is fairly large.

In contrast, in RCT-2, one-sixth of the patients benefit from treatment $A=a_{3}$ by being fully cured without any side-effects over being fully cured with some side-effects under treatment $A=a_{2}$. Although the proportion of population that benefits is $1/6$ in both cases, the benefit that can be ascribed to patients in RCT-1 is inarguably greater than that for patients in RCT-2.

Conceptually, with ordinal outcomes, we consider “small” benefits on an equal footing as “large” benefits, which leads to counter-intuitive conclusions and the intransitive ordering between treatments. Such problems do not occur when the outcomes are binary. In the binary case, there is no distinction between “small” or “large” benefits on the individual level: either an individual benefits or they do not. This is related to the fact that, in binary settings, interventionist decision rules coincide with rules based on the counterfactual criterion $P(\textrm{benefit}) \geq P(\textrm{harm})$.^2^^-^^4^ However, when potential harm and benefit on binary outcomes are assigned different weights,^3^^,^^8^ so-called asymmetric utility functions, the 2 notions of harm can give different decision rules.^2^^,^^5^^,^^8^^,^^9^

As opposed to binary outcomes, with ordinal outcomes we can encode our understanding of “small” and “large” benefits by the utility function $\mu $. With the particular utility function $\mu _{1}$, the difference in utility between having latent TB and being fully cured with some side-effects was $1$ unit, which was the same as the difference in utility between being cured with side-effects v/s being cured without any side-effects. We can tailor the utility function to reflect our perceived magnitude of benefits. In some settings, ordinal outcomes are simply binarized to survival and death, or to progression-free-survival and not. This implicitly imposes a certain utility function.

On the other hand, consider a utility function $\mu _{2}$ that maps to a number between $0$ and $10$; see Table 1. Death is the worst outcome, so it is assigned a utility of $0$. The various forms of TB are assigned utility between $3$ and $5$. Being fully cured with side-effects is not very different from being cured without side-effects, and both of these outcomes are much better than having TB in any form. Thus they are assigned utilities of $9$ and $10$ respectively. We can see that this utility function preserves the ordinal principle. Outcomes that are deemed better have higher utilities.

**Table 1:** Sample utility function $\mu _{2}.$

Outcome	$y_{1}$	$y_{2}$	$y_{3}$	$y_{4}$	$y_{5}$	$y_{6}$
Utility	0	3	4	5	9	10

Based on our selected utility function $\mu _{2}$, for the 3 treatments $A=a_{1},\ a_{2},$ and $a_{3},$ the expected utilities are $\mathbb{E}[\mu _{2}(Y^{a_{1}})] = 25/6$, $\mathbb{E}[\mu _{2}(Y^{a_{2}})] = 36/6$, and $\mathbb{E}[\mu _{2}(Y^{a_{3}})] = 30/6$. So we can say that ‘treatment with strong antibiotics’ $(A=a_{2})$ is better than both ‘treatment with weak antibiotics’ $(A=a_{3})$ and ‘no treatment’ $(A=a_{1})$ at maximizing utility.

However, this ordering is specific to our choice of utility function $\mu _{2}$. Consider another alternative utility function $\mu _{3}$ (Table 2) that is also order-preserving. The expected utilities are $\mathbb{E}[\mu _{3}(Y^{a_{1}})] = 40/6$, $\mathbb{E}[\mu _{3}(Y^{a_{2}})] = 30/6$, and $\mathbb{E}[\mu _{3}(Y^{a_{3}})] = 35/6$. The ordering of treatments with $\mu _{3}$ is the exact reverse of that achieved with $\mu _{2}$. It can be shown that any ordering between $a_{1}, a_{2},$ and $a_{3}$ is achievable with an appropriate order-preserving utility function.

**Table 2:** Sample utility function $\mu _{3}.$

Outcome	$y_{1}$	$y_{2}$	$y_{3}$	$y_{4}$	$y_{5}$	$y_{6}$
Utility	0	1	5	8	9	10

The selection of an appropriate utility function has already been richly discussed in the literature, eg, in the context of “utility elicitation” procedures.^28^^-^^30^ For example, in the health economics literature Quality Adjusted Life Years (QALYs) are often used as a measure of utility.^31^^-^^33^ Contributing to this debate is beyond the scope of this article.

## Conclusion

Applying counterfactual notions of harm holds the promise of improving decision making, eg, by minimizing the fraction of population negatively affected by treatment.^4^^,^^15^^,^^34^ However, the existing work predominantly compares only 2 alternatives for treatment, with some exceptions.^6^^,^^35^ Due to this restriction, they never encounter the problem of intransitivity highlighted here.

Intransitivity and harm have been discussed conceptually in the philosophy literature^35^^,^^36^ with qualitative arguments about comparing one treatment versus another. However, those works did not consider the definitions of counterfactual and interventionist harm (Definition 2.1 and 2.2).

Intransitivity can indicate that agents are being inconsistent or irrational in their choices and not objectively maximizing the desired outcome.^37^ Ordering treatments by counterfactual harm can lead to such intransitivity. On the other hand, the principle of minimizing interventionist harm produces a consistent transitive ordering once we have fixed a utility function.

Defining counterfactual harm requires consideration of joint counterfactuals which are fundamentally unobservable. We can only place bounds on the joint counterfactual distribution with data from a perfectly executed randomized experiment. Studies that seek bounds tighter than the assumption-free bounds^12^^,^^16^^,^^34^^,^^38^ employ assumptions on rank correlation between potential outcomes (individuals with higher outcomes under 1 treatment also have higher outcomes under the alternative treatment). Other studies on ordinal outcomes and the probability of benefit^14^^,^^17^ make strong monotonicity assumptions. As described,^12^^,^^16^^,^^17^ these bounds quickly become uninformative if rank correlation or monotonicity assumptions are relaxed. Such assumptions are also fundamentally untestable, that is, they are cross-world assumptions.^7^

Bounds might be sharpened by leveraging baseline covariates, which, eg, is a promising strategy in instrumental variable settings.^39^ Some success has been found in narrowing bounds when binary outcomes are considered; with increasing predictive power of the baseline covariates, the bounds on probability of benefit/harm might get tighter.^4^^,^^11^^,^^15^ However, the use of additional covariates does not guarantee point-identification of counterfactual harm. With nonbinary (continuous) outcomes, the improvement might be less promising; the bounds will in many cases remain uninformative even with covariate adjustment.^15^^,^^40^ Further, intransitivity in counterfactual harm is still a potential issue with nonbinary outcomes.

Lastly, we emphasize that our example was carefully constructed to exhibit intransitivity in the cleanest possible manner. The intransitivity holds for a wide class of joint distributions, including distributions satisfying rank correlation. In Appendix S3, we specify some instances of the full joint distribution for the TB example (*An example on TB and intransitivity* section) under rank correlation, demonstrating that intransitivity fails to hold only if outcomes are perfectly negatively correlated.

The conventional notion of counterfactual harm is inadequate as a decision criterion in nonbinary settings. If algorithmic decision rules are to be used in practice, such as in clinical medicine, they must account for the possibility of multiple treatment or intervention options. Our results show that investigators should be careful when defining harm and their utility functions.

## Supplementary Material

Web_Material_kwag064

## Data Availability

The article is purely theoretical and uses no data.
