# Supplementary material for: Counterfactual harm: a counter-argument
Source: Am J Epidemiol. 2026 Mar 19;195(8):2230–6. doi: 10.1093/aje/kwag064 (PMC13437087; doi:10.1093/aje/kwag064)
Supplement: Supplementary file 1 [file web_material_kwag064.zip › Web_Material_kwag064.pdf]

American Journal of Epidemiology **Supplementary Material**[1]

Special Collection: N/A.

Title: Counterfactual Harm: A Counter-argument

Authors: Amit N. Sawant, Mats J. Stensrud

Table of Contents:

Appendix S1: Mathematical derivation of bounds from RCT-1

Appendix S2: Weighted harm and benefit

Appendix S3: Rank correlated joint distributions

[1] N/A indicates not applicable.

S1. Mathematical derivation of bounds from RCT-1

To derive bounds on $P(Y^{a_2} > Y^{a_1})$ consider the following four principal stratum probabilities:

$$
\begin{aligned}
P(Y^{a_1} = y_1,\ Y^{a_2} = y_2) &= p_1,\\
P(Y^{a_1} = y_1,\ Y^{a_2} = y_5) &= p_2,\\
P(Y^{a_1} = y_4,\ Y^{a_2} = y_2) &= p_3,\\
P(Y^{a_1} = y_4,\ Y^{a_2} = y_5) &= p_4.
\end{aligned}
$$

We know that $p_1,\ p_2,\ p_3,\ p_4 \geq 0$ and $p_1 + p_2 + p_3 + p_4 = 1$. Additionally, from the observed marginals in RCT-1, we can impose the following constraints,

$$
\begin{aligned}
&P(Y^{a_1} = y_1) = p_1 + p_2 = 1/6 &&\Rightarrow p_1 \leq 1/6,\\
&P(Y^{a_2} = y_2) = p_1 + p_3 = 1/2 &&\Rightarrow p_3 = 1/2 - p_1,\\
&P(Y^{a_2} = y_5) = p_2 + p_4 = 1/2.
\end{aligned}
$$

The probability of benefit is given by

$$P(Y^{a_2} > Y^{a_1}) = p_1 + p_2 + p_4 = 1/2 + p_1, \quad (0 \leq p_1 \leq 1/6)$$

and respectively, the probability of counterfactual harm,

$$P(Y^{a_2} < Y^{a_1}) = p_3 = 1/2 - p_1. \qquad (0 \leq p_1 \leq 1/6)$$

These expressions give us the bounds from RCT-1 (Figure 2). Furthermore, $P(Y^{a_2} > Y^{a_1}) = P(Y^{a_2} < Y^{a_1}) = 1/2$ occurs only if $p_1 = 0$, i.e. there are no patients belonging to the principal stratum $(Y^{a_1} = y_1,\ Y^{a_2} = y_2)$. Thus, strong antibiotics are strictly more beneficial than harmful compared to no treatment, except if there are strong determinisms in the data.

The bounds from RCT-2 can be derived in an identical manner. In RCT-2, the edge case of $P(Y^{a_3} > Y^{a_2}) = P(Y^{a_3} < Y^{a_2}) = 1/2$ occurs only if $P(Y^{a_2} = y_5,\ Y^{a_3} = y_6) = 0$, otherwise weak antibiotics are strictly more beneficial than harmful compared to strong antibiotics.

S2. Weighted harm and benefit

In some literature on counterfactual harm, the decision rule uses a weighted linear combination of benefit and harm [1, 5, 6]. The gain if an individual benefits is denoted by u_g, and the loss if an individual is harmed is $-u_l$, where $0 < u_g \leq u_l$ [1]. The greater loss in case the individual is harmed reinforces the principle of loss-aversion or "do no harm". Notably, this consideration of an asymmetric decision rule can result in interventionist and counterfactual rules differing in even binary settings [1, 5].

The weighted decision rule $u_g \cdot P(\text{benefit}) - u_l \cdot P(\text{harm}) \geq 0$, or equivalently $P(\text{benefit}) - w \cdot P(\text{harm}) \geq 0$ where $w = u_l/u_g$, gives different treatment recommendations for different values of w. We apply such a rule to the examples of RCT-1 and RCT-2. For our particular examples, the probability of the outcome being exactly the same with both treatment options is zero, i.e. benefit and harm are complementary events with $P(\text{benefit}) + P(\text{harm}) = 1$. As a result, the decision rule $P(\text{benefit}) - w \cdot P(\text{harm}) \geq 0$ can be rewritten as $P(\text{benefit}) \geq w/(1 + w)$.

In RCT-1 (Section 3.2) we compared 'Strong Antibiotics' ($A = a_2$) to 'No Treatment' ($A = a_1$) and obtained the bounds

$$
\begin{aligned}
1/2 \leq P(\text{benefit}; a_2, a_1) &\leq 2/3,\\
1/3 \leq P(\text{harm}; a_2, a_1) &\leq 1/2.
\end{aligned}
$$

If $w = 1$, we could conclusively say that benefit was greater than (or equal to) harm. If we choose a value of $w \in (1, 2)$, it is possible that the expected counterfactual utility is positive with the given bounds, but we cannot conclusively say so. For $w > 2$, the expected counterfactual utility is always negative.

In RCT-2, we derived the same bounds when comparing 'Weak Antibiotics' to 'Strong Antibiotics'. For benefit and harm in RCT-2

$$
\begin{aligned}
1/2 \leq P(\text{benefit}; a_3, a_2) &\leq 2/3,\\
1/3 \leq P(\text{harm}; a_3, a_2) &\leq 1/2.
\end{aligned}
$$

Thus, the decision of which treatment among strong or weak antibiotics is the "better" one will depend on w in the same manner as it did in RCT-1.

It is debatable whether the value of w selected for RCT-1 should be the same as that for RCT-2. If we select the same value of w for both comparisons, we are being consistent with our decision rule. However, it leads to the same problem that "small" harms in RCT-1 receive the same weight as "big" harms in RCT-2.

S3. Rank correlated joint distributions

In the Conclusion section, we commented that the intransitive order between TB treatments occurs for a wide class of joint distributions, including distributions satisfying rank correlation. We expand on the statement in this Appendix.

First we describe rank correlation conceptually. The term "rank correlation" is used to indicate that the joint potential outcomes are correlated. Suppose that an individual has an outcome higher than the population average under treatment $A = a_1$, thereby having a higher rank amongst all individuals in the population. Conceptually, if this individual's outcome under treatment $A = a_2$ would be expected to be higher than the population average under treatment $A = a_2$, then we say that the joint potential outcomes are positively rank correlated. Rank correlation can indicate the plausible assumption that healthier individuals are more likely to survive under no treatment, and are also more likely to respond to treatment and thus get cured. So joint outcomes are positively correlated.

To fix ideas, consider an individual with the best possible outcome under no treatment who can have a better-than-average, but not the best, outcome under treatment. Such a scenario still represents positive rank correlation, but not exact correlation.

The assumption of strong positive rank correlation is sometimes invoked to sharpen bounds on potential benefit/harm [2–4, 7]. For instance, Ding et al. [3] commented that the maximum of potential harm occurs when joint outcomes are perfectly negatively associated, which is unlikely to happen in practice. Similarly, Wu et al. [7] commented that a patient's unmeasured health status can affect potential outcomes under both treatment and control, making the joint outcomes positively correlated. Therefore, bounds on potential harm can be sharpened by assuming joint outcomes are positively rank correlated.

In Section 3, we described only the marginal distributions of outcomes, without making any additional assumptions on the joint

distribution of potential outcomes under different treatments. We now exactly specify two joint distributions of the example from Section 3. First, we corroborate Ding et al. [3], showing that the the edge case where $P(\text{benefit}) = P(\text{harm}) = 1/2$ is attained precisely when joint outcomes are perfectly negatively correlated.

In RCT-1, we compared outcomes under no treatment ($A = a_1$) and under strong antibiotics ($A = a_2$). Consider the one-sixth of individuals that die of TB ($Y^{a_1} = y_1$). Such individuals are ranked the lowest in the population in terms of outcomes under no treatment. If joint outcomes are negatively rank correlated, these individuals tend to be cured with side-effects, the best possible outcome when given strong antibiotics ($Y^{a_2} = y_5$).

The joint potential outcomes follow the law $P(Y^{a_1} = y_1, Y^{a_2} = y_5) = 1/6$, $P(Y^{a_1} = y_1, Y^{a_2} = y_2) = 0$, which is depicted in the right set of pies in Figure 2. Such a distribution corresponds to the maximum potential harm $P(Y^{a_2} > Y^{a_1}) = 1/2$.

Similarly, in the right set of pies in Figure 3, the upper bound for harm, $P(Y^{a_3} > Y^{a_2}) = 1/2$, is attained only if outcomes satisfy the law $P(Y^{a_2} = y_2, Y^{a_3} = y_6) = 1/6$, $P(Y^{a_2} = y_5, Y^{a_3} = y_6) = 0$. In this scenario, the individuals with the best possible outcome under weak antibiotics (y_6 : Cured without side effects) always have the worst possible outcome under strong antibiotics (y_2 : XDR-TB), meaning outcomes are negatively rank correlated.

Negative rank correlation is theoretically possible, but has been argued to be implausible [3, 7]. Thus, the equality $P(\text{benefit}) = P(\text{harm}) = 1/2$ only happens in an arguably contrived special case.

Next, we specify the joint distribution with positive rank correlation. Take the most healthy individuals who have the best possible outcomes under each treatment. For these individuals the joint outcomes are $P(Y^{a_1} = y_4, Y^{a_2} = y_5, Y^{a_3} = y_6) = 1/6$. Next, the slightly less healthier individuals do not get cured with weak antibiotics, but do respond to strong antibiotics $P(Y^{a_1} = y_4, Y^{a_2} = y_5, Y^{a_3} = y_3) = 1/3$. The other individuals are divided as $P(Y^{a_1} = y_4, Y^{a_2} = y_2, Y^{a_3} = y_6) = 1/3$ and $P(Y^{a_1} = y_1, Y^{a_2} = y_2, Y^{a_3} = y_3) = 1/6$. Thus, the weakest individuals are the ones that would die under no treatment, get XDR-TB under strong antibiotics, and get MDR-TB when treated with weak antibiotics.

Given this joint distribution of outcomes, we have that $P(Y^{a_2} > Y^{a_1}) = P(Y^{a_3} > Y^{a_2}) = P(Y^{a_1} > Y^{a_3}) = 2/3$ and the treatments are maximally intransitive. We conclude that the assumption of positive rank correlation, which has been invoked to sharpen bounds on potential benefit/harm [4, 7], does not guarantee transitivity of counterfactual harm comparisons. In fact, even with perfect knowledge of the joint counterfactual distribution, we cannot discount the possibility of intransitivity.

References

1. Eli Ben-Michael, Kosuke Imai, and Zhichao Jiang. Policy learning with asymmetric counterfactual utilities. *Journal of the American Statistical Association*, 119(548):3045–3058, 2024.
2. Yifan Cui. Individualized decision-making under partial identification: Three perspectives, two optimality results, and one paradox. *arXiv preprint arXiv:2110.10961*, 2021.
3. Peng Ding, Avi Feller, and Luke Miratrix. Decomposing treatment effect variation. *Journal of the American Statistical Association*, 114(525):304–317, 2019.
4. Michael Gechter. Generalizing the results from social experiments: Theory and evidence from india. *Journal of Business & Economic Statistics*, 42(2):801–811, 2024.
5. Andrew Gelman and Jonas M Mikhaeil. Russian roulette: the need for stochastic potential outcomes when utilities depend on counterfactuals. *Biometrika*, 112(4):asaf062, 2025.
6. Jonathan Richens, Rory Beard, and Daniel H Thompson. Counterfactual harm. *Advances in Neural Information Processing Systems*, 35:36350–36365, 2022.
7. Peng Wu, Peng Ding, Zhi Geng, and Yue Liu. Quantifying individual risk for binary outcome. *arXiv preprint arXiv:2402.10537*, 2024.
